# APOA1 mRNA and protein in kidney renal clear cell carcinoma correlate with the disease outcome

**DOI:** 10.1038/s41598-022-16434-6

**Published:** 2022-07-20

**Authors:** Wei Zeng, Guoguang Xiong, Li Hua, Yugang Hu, Xufeng Guo, Xiulan Peng

**Affiliations:** 1 Department of Neurology, The Second Affiliated Hospital of Jianghan University, Wuhan, 430000 Hubei Province People’s Republic of China; 2 Department of Urology, The Second Affiliated Hospital of Jianghan University, Wuhan, 430050 Hubei Province People’s Republic of China; 3 Department of General Medicine, The Second Affiliated Hospital of Jianghan University, Wuhan, 430050 Hubei Province People’s Republic of China; 4 Department of Ultrasonography, Renmin Hospital of Wuhan University, Wuhan, 430060 Hubei Province People’s Republic of China; 5 Department of Oncology, Renmin Hospital of Wuhan University, Wuhan, 430060 Hubei Province People’s Republic of China; 6 Department of Oncology, The Second Affiliated Hospital of Jianghan University, 122 Xianzheng Road, Wuhan, 430050 Hubei Province People’s Republic of China

**Keywords:** Cancer, Biomarkers, Medical research, Nephrology, Oncology, Risk factors

## Abstract

Renal cancer is one of the most common malignant tumors with high mortality, and kidney renal clear cell carcinoma (KIRC) is the most common type of renal cancer. We attempted to evaluate the clinical and prognostic significance of Apolipoprotein A1 (APOA1) mRNA and protein in KIRC patients. Clinical data along with RNA-sequencing data were downloaded from UCSC Xena. The Human Protein Atlas database was searched to reveal APOA1 protein expression profiles in KIRC and normal renal tissues. The TIMER database was applied to determine the correlations of APOA1 with immune cells and PD-1 and PD-L1 in KIRC. Ninety-one cases of KIRC patients and 93 healthy controls from our hospital were enrolled for clinical validation. Levels of APOA1 mRNA in KIRC tissues (N = 535) are not only lower than the levels in normal renal tissues (N = 117), but also in paired normal renal tissues (N = 72). High expression of APOA1 mRNA at the time of surgery was correlated with worse overall survival (OS) (HR 1.66; *p* = 0.037) and disease-free survival (DFS) (HR 1.65; *p* = 0.047), and APOA1 DNA methylation was linked to worse OS (HR 2.1; *p* = 0.001) rather than DFS (HR 1.12; *p* = 0.624) in KIRC patients. Concentrations of preoperative serum APOA1 protein were markedly decreased in KIRC patients compared to healthy controls (*p* < 0.01), and low levels of APOA1 protein predicted less favorable OS than those with high levels (HR = 2.84, *p* = 0.0407). APOA1 negatively correlated with various immune cell infiltrates and PD-L1 expression (r = − 0.283, *p* = 2.74e−11) according to the TIMER database. Low levels of APOA1 mRNA at the time of surgery predict favorable survival in KIRC patients. Our results provide insights to identify a novel prognostic index with great clinical utility.

## Introduction

The American Cancer Society predicted that 73,750 people in the United States would be newly diagnosed with renal carcinoma by the end of 2020^1^. The most common subtype of renal cancer is kidney renal clear cell carcinoma (KIRC), which is also the most common malignancy of the urinary system^2^. For patients with KIRC and only local invasion, surgical resection remains the primary and most effective treatment. However, in patients with KIRC who have remote metastases, patients with local KIRC tumors that have been excised by nephrectomy are still at high risk of recurrence and are insensitive to radiotherapy or chemotherapy^2^, leading to poor prognoses. The development of targeted therapeutics, including mTOR inhibitors and multitargeted tyrosine kinase inhibitors, has emerged as a breakthrough in the treatment of KIRC. Recently, immunotherapy has been viewed as an effective treatment option against KIRC with advanced TNM stage^3^. Therefore, determining the appropriate markers for survival prediction is essential for the treatment and prognosis of patients with KIRC.

Although KIRC has been extensively studied, knowledge of its molecular mechanisms and accurate predictions about the prognosis is limited. KIRC exhibits significant molecular heterogeneity^4^ that involves multiple changes in gene expression. With the help of bioinformatics, a series of key genes associated with renal cancer have been identified as potential biomarkers for diagnosis or survival prediction and as therapeutic targets^5–7^. However, since most of these biomarkers are not measured routinely in clinical practice, they are not clinically useful. Therefore, identifying a novel prognostic marker that is easily accessible clinically and that can accurately predict survival is essential for the management of patients with KIRC.

Apolipoprotein A1 (APOA1), a common index of blood lipids, is believed to play an essential role in the occurrence and progression of cardiovascular diseases^8–10^. Recently, APOA1 has been associated with survival in patients with colorectal^11,12^, ovarian^13^, breast^14^, and liver cancer^15^. Moreover, two studies have demonstrated that preoperative APOA1 concentration is an independent factor affecting survival in patients with metastatic renal cell cancer^16,17^. Zhang et al.^16^ also revealed the ratio of serum APOB to APOA1 as a potent risk factor in renal cell carcinoma, and that the lower ratio is associated with better survival. Moreover, Guo et al.^17^ proved that elevated serum APOA1 concentration is related to better survival outcomes in patients with KIRC. However, no study has comprehensively evaluated the association between APOA1 mRNA and its protein and primary KIRC. In this study, we applied bioinformatics to explore the levels of APOA1 mRNA, methylation, and protein in terms of their expression and prognosis in patients with KIRC. We also collected clinical data from 91 patients with KIRC with follow-up data from our hospital to investigate the prognostic role of the serum APOA1 protein in KIRC. We investigated the clinical significance of APOA1 mRNA and protein, which is routinely detected in clinical practice. Our study is a good example of bioinformatics in combination with clinical data, which is different from the purest bioinformatic analysis.

## Methods

### UCSC Xena

UCSC Xena (http://xena.ucsc.edu/#overview) is an online exploration website for visualization and analysis of cancer genomic data based on The Cancer Genomic Atlas (TCGA) database^18^, which is publicly available to all users. In this study, we extracted APOA1 expression data from 535 patients with KIRC and 72 subjects with normal renal tissue using the visualization module. The data set module was used to download clinical information from the TCGA-KIRC dataset. Subsequently, the visualization module was used again to determine any association between APOA1 mRNA and APOA1 DNA methylation.

### TNMplot database

The TNMplot database^19^ is an online platform (https://tnmplot.com/analysis/) for the analysis of differential gene expression in normal, tumor, and metastatic tissues. This database contains 56,938 multilevel quality-controlled samples from TCGA, Therapeutically Applicable Research to Generate Effective Treatments (TARGET), GEO, and Genotype-Tissue Expression repositories. Importantly, this platform provides unique metastatic data. We used this online platform to investigate the expression of APOA1 in KIRC and normal tissues.

### LinkedOmics

The LinkedOmics website (http://www.linkedomics.org/) is an easily accessible research platform that provides a series of data analyses based on the TCGA dataset^20^. In our study, we searched this database to identify the genes most significantly associated with APOA1 expression in KIRC tissue through the LinkFinder module and then selected these genes for enrichment analysis using the LinkerInterpreter module and the Gene Set Enrichment Analysis (GSEA) tool.

### Human Protein Atlas (HPA)

The HPA database (https://www.proteinatlas.org/) is an open-source database of all types of human proteins found in normal and cancerous tissues^21^. In the current study, the protein expression module was used to investigate the protein levels of APOA1 in normal renal tissues and KIRC tissues, as detected by immunohistochemistry in the HPA database. Two anti-APOA1 antibodies (HPA046715 and CAB016778) were used to determine the levels of APOA1 protein expression in normal renal and KIRC tissues.

### TIMER database

The TIMER database (https://cistrome.shinyapps.io/timer/) is a valuable resource for a comprehensive analysis of immune infiltrates in various types of cancer^22^. This database provided us with six immune infiltrates estimated by the TIMER algorithm. The correlation between APOA1 expression and infiltrating immune cells was detected using the gene module, and the correlation module was used to determine the association between *APOA1* and other genes (*PD-1/PD-L1*). The correlation module was then used to determine the association between APOA1 and immune cell markers.

### UALCAN

UALCAN is a very friendly web tool (http://ualcan.path.uab.edu/index.html) that can be used to accurately analyze omics data^23^. In this study, we used the UALCAN tool to analyze the differential expression of APOA1 mRNA in KIRC tissue compared to normal renal specimens. To do this, we entered the APOA1 gene into the TCGA gene analysis module, selected KIRC, and then obtained the differential expressions of APOA1 both in KIRC tissues and in normal specimens.

### Clinical data from the RHWU cohort

We retrospectively enrolled 91 patients diagnosed with KIRC treated at RHWU between January 2016 and December 2019. An additional 93 healthy individuals were enrolled as controls. The study outline was reviewed and smoothly approved by the ethics committee of the RHWU (No 2017-CK107). Written informed consent was obtained from all patients before enrollment in the study. Blood from patients with KIRC was collected 1 week before surgery. Based on the standardized operation, serum APOA1 concentrations were automatically detected using an autoanalyzer (Siemens ADVIA 2400, Germany). We also obtained other clinical data, such as sex, age at diagnosis, tumor grade, tumor site, T stage, N stage, M stage, and TNM stage, from electronic medical records. Follow-up was conducted by telephone interview until August 31, 2021. Overall survival (OS) was calculated as the interval between surgical resection of the renal tumor and the last follow-up or death from any cause.

### Statistical analysis

Continuous and categorical variables are expressed as means with standard deviations and numbers with percentages, respectively. For categorical variables, differences between the two groups were analyzed using the chi-square or Fisher’s exact test. For continuous variables, differences were determined using the Student’s *t*-test or nonparametric test. Receiver operating characteristic (ROC) curves were drawn to assess APO1 mRNA and serum protein levels as potential diagnostic predictors. Kaplan–Meier curves were plotted to assess APOA1 mRNA and protein levels as prognostic indicators, and the log-rank test was used to assess the difference in survival between the two groups. Cox regression analyses were performed to determine whether APOA1 mRNA or APOA1 protein levels were independent biomarkers of prognosis in patients with KIRC. The correlation between the two genes was determined using the Spearman correlation test. All statistical analyses were performed using SPSS 20.0 (https://www.ibm.com/cn-zh/analytics/spss-statistics-software). Graphpad Prism (version 9.0, https://www.graphpad.com/scientific-software/prism/) was utilized to generate the survival curves, correlation plots, ROC curves and comparison plots. All tests were performed on both sides, and a *p-*value < 0.05 was considered statistically significant.

### Ethics approval

The study outline was reviewed and smoothly approved by the ethics committee of the Renmin Hospital of Wuhan University (No 2017-CK107). All patients provided written informed consent prior to enrollment in the study. All methods were carried out in accordance with relevant guidelines and regulations.

## Results

### APOA1 mRNA expression and DNA methylation in KIRC

**Figure 1:**
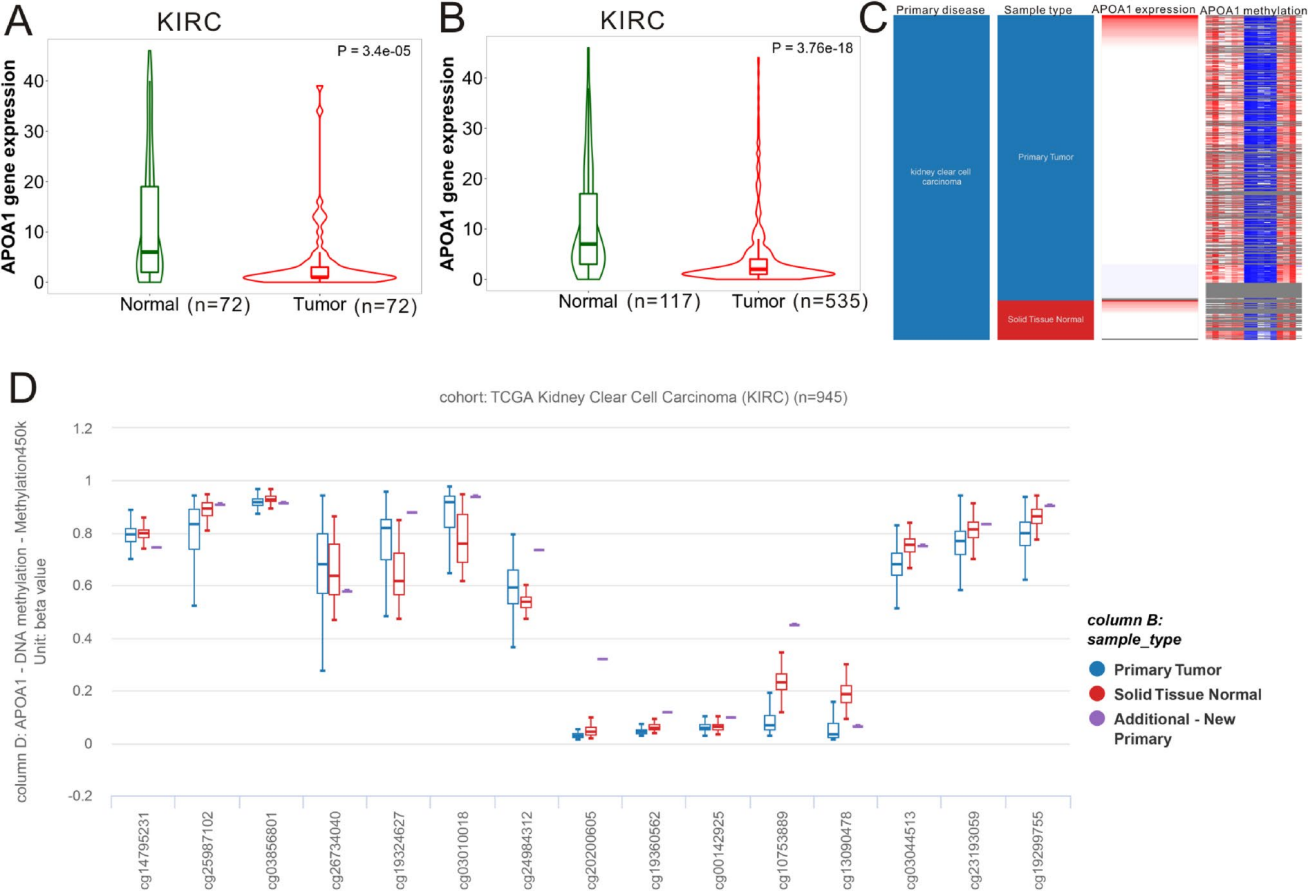
Expression and methylation of APOA1 in KIRC tissues and the normal renal tissues. (**A**) The levels of APOA1 mRNA in KIRC tissues were statistically lower than that in paired normal renal tissues (*p* = 3.4e−5) by TNMplot (https://tnmplot.com/analysis/). (**B**) The levels of APOA1 mRNA in KIRC tissues were statistically lower than that in the normal renal tissues (*p* = 3.76e−18) by TNMplot (https://tnmplot.com/analysis/). (**C**) UCSC Xena webpage (http://xena.ucsc.edu/#overview) was browsed to generate the heatmap of APOA1 mRNA expression and DNA methylation in KIRC dataset. (**D**) Distribution of APOA1 DNA CpG site among primary tumors, normal tissues and additional-new primary tumors was obtained from UCSC Xena webpage (http://xena.ucsc.edu/#overview).

The TNMplot web tool was used to analyze the expression profiles of APOA1 mRNA in KIRC tissues compared with that in normal tissues. The results (Fig. 1A) indicated that the levels of APOA1 mRNA in KIRC tissues were statistically lower than those of the paired normal renal tissues (*p* = 3.4e−5). Regarding non-paired comparison, we also observed that APOA1 mRNA levels in KIRC tissues were statistically lower than in normal renal tissues (*p* = 3.76e−18, Fig. 1B). We then extracted GSE53757 to validate the differential expression of APOA1 mRNA between KIRC tissues and normal renal tissues and obtained a similar conclusion (Fig. S1A). The ROC curve was used to assess the potential role of APOA1 as a diagnostic marker in patients with KIRC. As shown in Fig. S1B, APOA1 was acceptable for distinguishing KIRC tissue from normal tissue according to TCGA-KIRC data, with an area under the curve (AUC) of 0.708 (95% CI 0.647–0.769). We measured the diagnostic ability of APOA1 mRNA based on GSE53757, and the AUC was 0.8279 (95% CI 0.7581–0.8977, Fig. S1C). The UCSC Xena tool was then used to study the association between APOA1 expression and DNA methylation in KIRC tissue. As shown in Fig. 1C, a weak association was observed between APOA1 expression and DNA methylation. A correlation analysis (Table S1) was performed to find any association between APOA1 expression and certain APOA1 DNA CpG sites. Three APOA1 CpG sites (cg25987102, cg00142925, and cg19299755) were significantly associated with APOA1 expression (Fig. S2). The distribution plot of APOA1 DNA CpG sites also showed that the levels of APOA1 methylation between KIRC tissues and normal tissues were significantly different (Fig. 1D). A chi-square analysis revealed that the expression of APOA1 mRNA was significantly associated with histological grade (*p* = 0.042) and white blood cell count (*p* = 0.047) (Table S2). Similarly, APOA1 DNA methylation levels were markedly correlated with the N stage (*p* = 0.031), M stage (*p* = 0.001), and TNM stage (*p* = 0.045), indicating that APOA1 DNA methylation may be a novel indicator of metastasis in patients with KIRC.

### Prognostic role of APOA1 mRNA in KIRC

**Figure 2:**
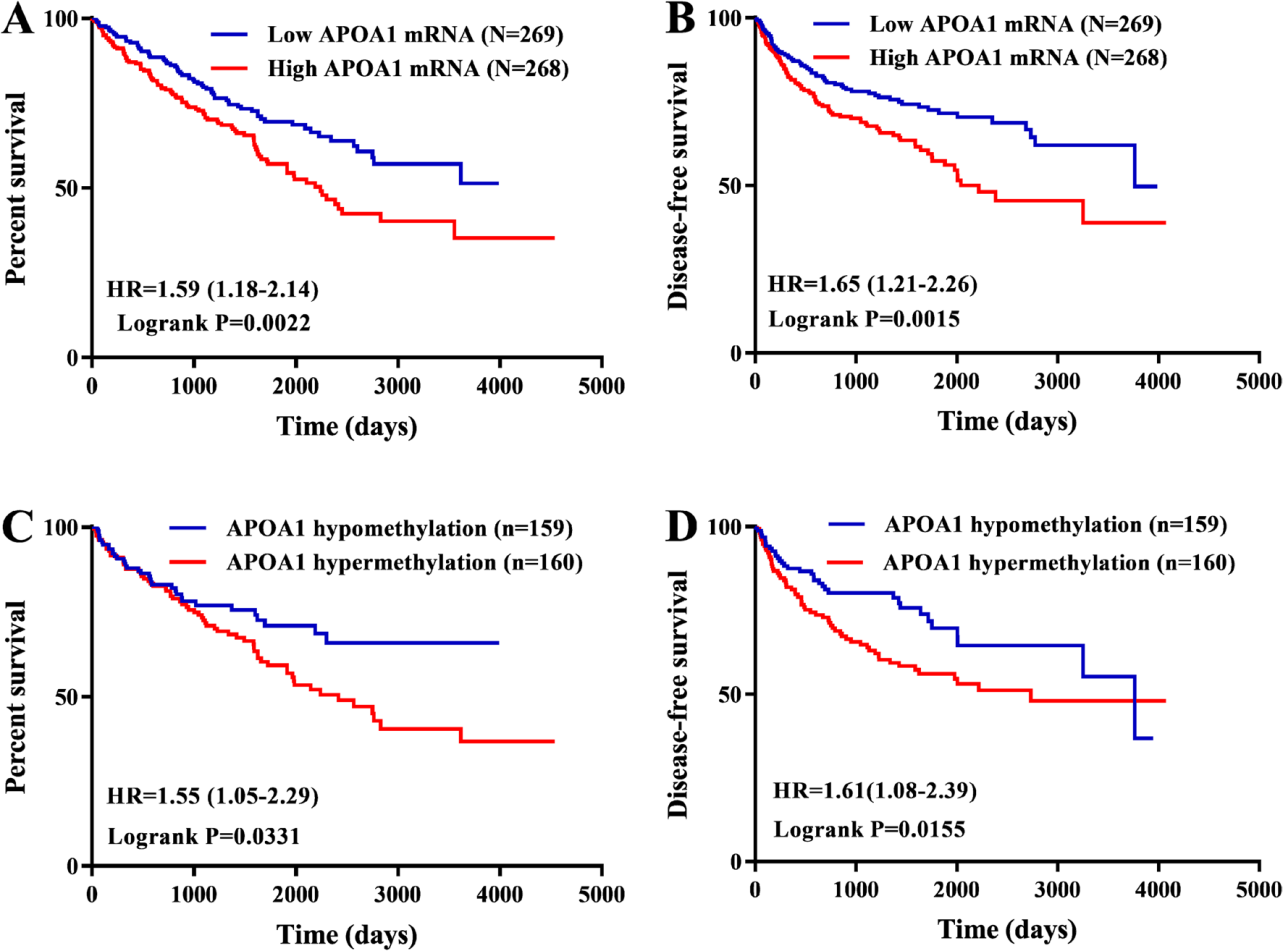
Survival analyses comparing low and high APOA1 mRNA/methylation in KIRC patients by Grahpad Prism version 9.0 (https://www.graphpad.com/scientific-software/prism/). KIRC patients with low levels of APOA1 mRNA (N = 269) exhibited longer overall survival (**A**) and disease-free survival (**B**) time than those with high levels of APOA1 mRNA (N = 268). KIRC patients with APOA1 hypomethylation (N = 159) exhibited longer overall survival (**C**) and disease-free survival (**D**) time than those with APOA1 hypermethylation (N = 160).

Survival analyses revealed the prognostic significance of APOA1 mRNA and APOA1 DNA methylation for KIRC. Kaplan–Meier curves showed that low expression of APOA1 mRNA was associated with favorable OS (HR 1.59; *p* = 0.0022) and disease-free survival (DFS) (HR 1.61; *p* = 0.015) (Fig. 2). Similarly, survival analyses also demonstrated that APOA1 DNA hypermethylation was correlated with unfavorable OS (HR 1.55; *p* = 0.0331) and DFS (HR 1.61; *p* = 0.015). Cox regression analyses were then performed to identify independent prognostic indicators for KIRC. The results showed that high expression of APOA1 mRNA was a reliable marker of unfavorable OS (HR 1.66, *p* = 0.037) and DFS (HR 1.65, *p* = 0.047), and APOA1 DNA hypermethylation was a reliable biomarker of unfavorable OS (HR 2.1, *p* = 0.001) but not a significant biomarker for DFS (HR 1.12, *p* = 0.624) (Table 1). In summary, APOA1 mRNA was determined to be a reliable indicator for predicting survival (OS and DFS) among patients with KIRC, while APOA1 DNA methylation may be a novel biomarker for predicting OS.

**Table 1:** Multivariable analysis of APOA1 mRNA expression/methylation for overall survival/disease-free survival.

Variables	Overall survival			Disease-free survival		
	HR	95% CI	*p* value	HR	95% CI	*p* value
Age (years)						
≥ 50	1.74	1.09–2.78	0.021			
< 50	Ref	–	–			
Gender						
Male				1.22	0.77–1.94	0.403
Female				Ref	–	–
Laterality						
Left	1.56	0.99–2.44	0.053	1.82	1.19–2.80	0.006
Right	Ref	–	–	Ref	–	–
Longest dimension						
≥ 1.5 cm	1.07	0.69–1.69	0.755			
< 1.5 cm	Ref	–	–			
Histologic Grade						
G1 + G2	Ref	–	–	Ref	–	–
G3 + G4	1.72	1.00–2.98	0.052	2.45	1.44–4.17	0.001
T stage						
T1 + T2	Ref	–	–	Ref	–	–
T3 + T4	1.86	0.80–4.34	0.152	1.18	0.61–2.29	0.627
N stage						
N0 + NX	Ref	–	–	Ref	–	–
N1	6.50	2.24–18.9	0.001	2.83	0.93–8.59	0.066
M stage						
M0 + MX	Ref	–	–	Ref	–	–
M1	3.07	1.71–5.450	< 0.001	3.43	2.04–5.76	< 0.001
TNM stage						
I + II	Ref	–	–	Ref	–	–
III + IV	1.69	0.92–3.10		5.06	2.15–11.87	< 0.001
Radiation						
Yes				3.65	0.82–16.36	0.091
No				Ref	–	–
Neoadjuvant treatment						
Yes	1.10	0.32–3.82	0.877			
No	Ref	–	–			
APOA1expression						
Low	Ref	–	–	Ref	–	–
High	1.66	1.05–2.62	0.031	2.10	1.34–3.28	0.001
APOA1 methylation						
Low	Ref	–	–	Ref	–	–
High	1.65	1.01–2.70	0.047	1.12	0.72–1.74	0.624

### Expression of the APOA1 protein based on the HPA database

**Figure 3:**
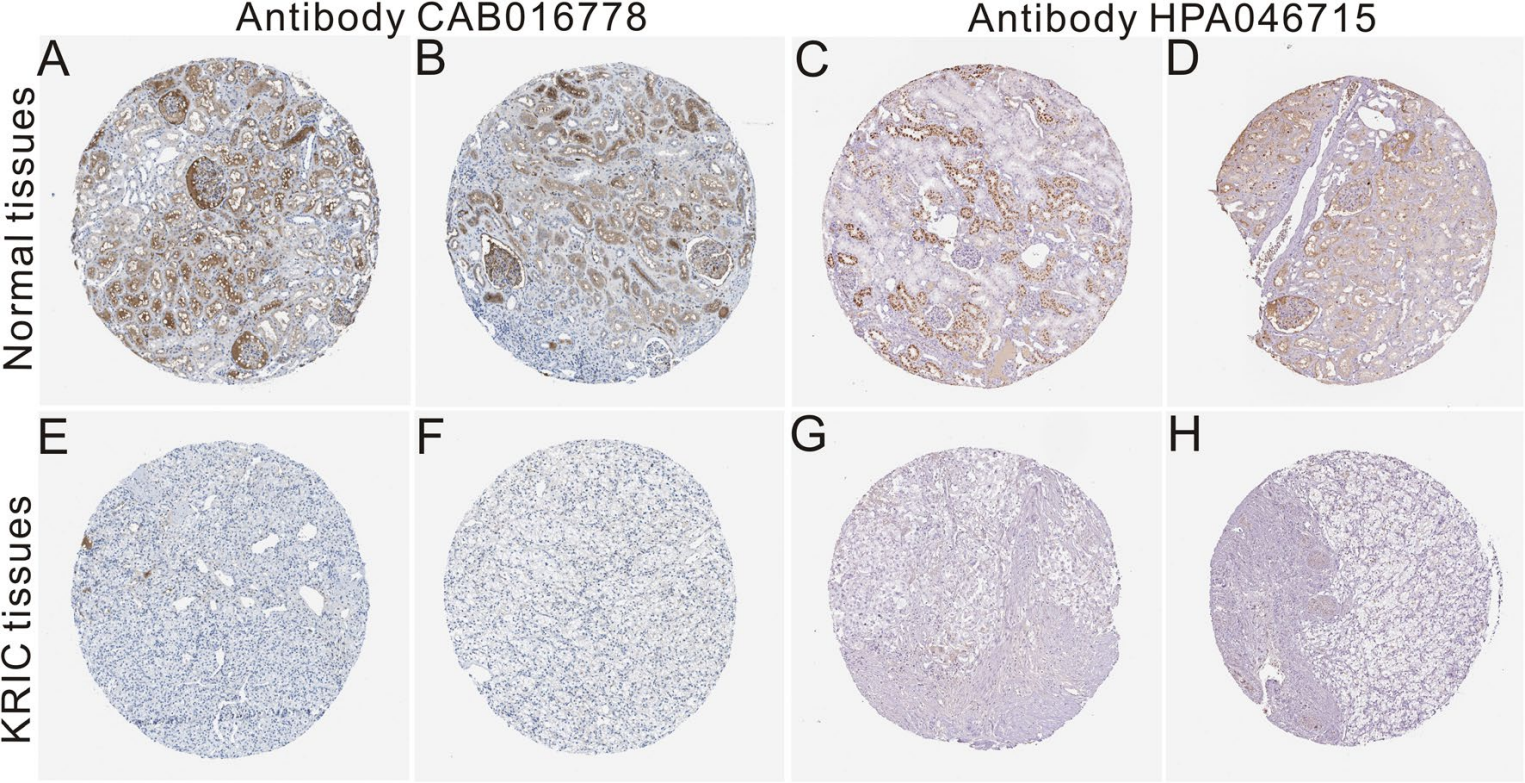
Representative images stained with APOA1 antibody (CAB016778; HPA046715), which were downloaded from the HPA database (https://www.proteinatlas.org/). (**A**–**D**) The normal tissues. (**E**–**H)** KIRC tissues. HPA046715 is a polyclonal antibody derived from rabbits and produced by Sigma-Aldrich, and CAB016778 is a monoclonal antibody derived from mice and produced by BioPorto Diagnostics A/S.

The HPA database was systematically searched to analyze APOA1 protein expression patterns in cancerous tissues compared to normal renal tissues. Two types of APOA1 antibodies (CAB016778 and HPA046715) were used for immunohistochemistry. Three cases of normal renal tissue were strongly stained with the two antibodies. However, 12 of the cases of renal cancerous tissues were not positive for CAB016778, and only one case showed weak positive staining for HPA046715. Representative images stained with the APOA1 antibody are shown in Fig. 3A–H. We concluded that APOA1 protein expression was downregulated in renal cancerous tissues compared to that in normal renal tissues.

### Levels of serum APOA1 protein in patients with KIRC

**Figure 4:**
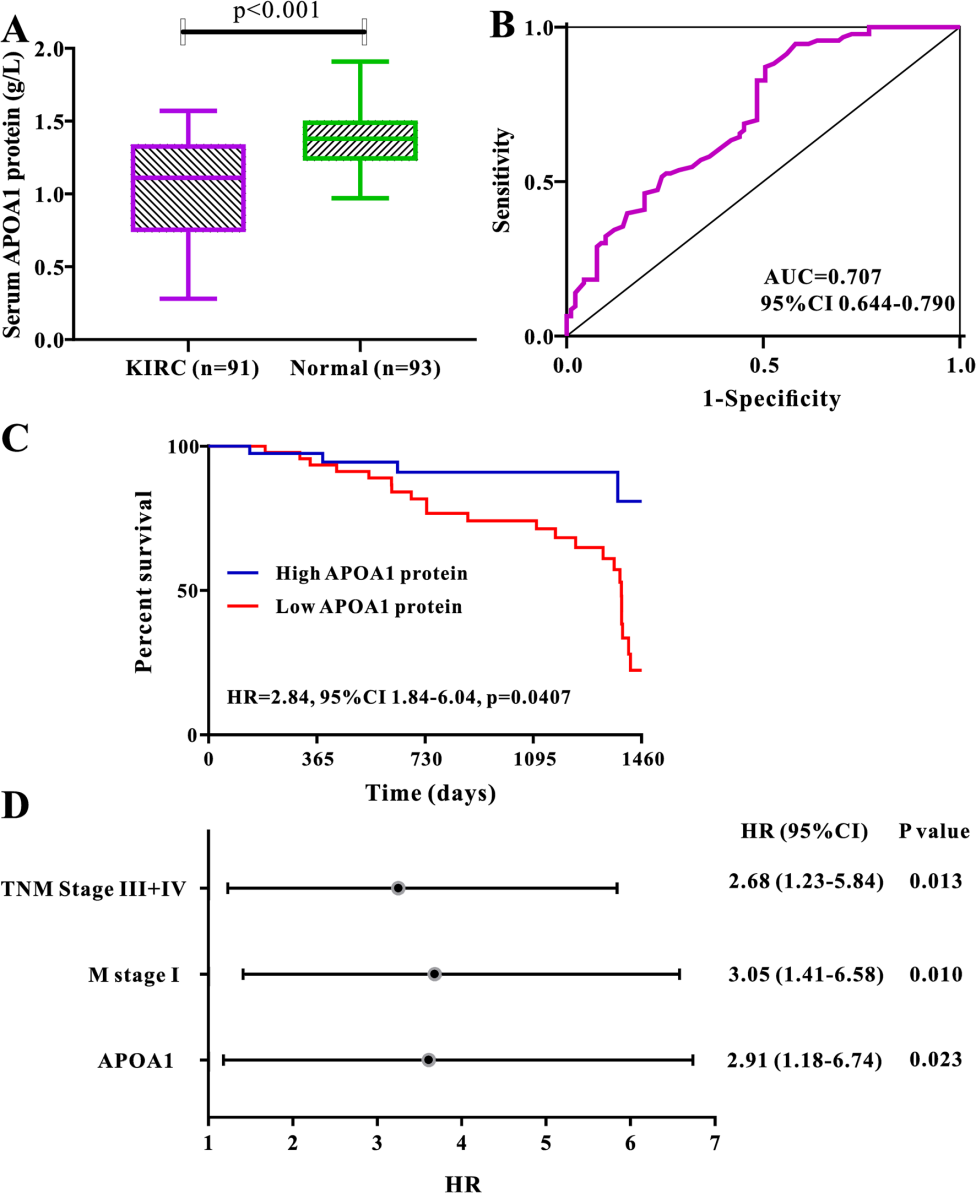
Diagnostic and prognostic significance of serum APOA1 protein in KIRC patients by Grahpad Prism version 9.0 (https://www.graphpad.com/scientific-software/prism/). (**A**) Levels of serum APOA1 protein in healthy individuals are much higher than that in KIRC patients (*p* < 0.001). (**B**) ROC curve of serum APOA1 protein for the identification of KIRC patients from normal controls. (**C**) Kaplan–Meier plot of serum APOA1 protein among 91 cases of KIRC patients. (**D**) Forest plot of the multivariable analysis of overall survival in KIRC patients.

**Figure 5:**
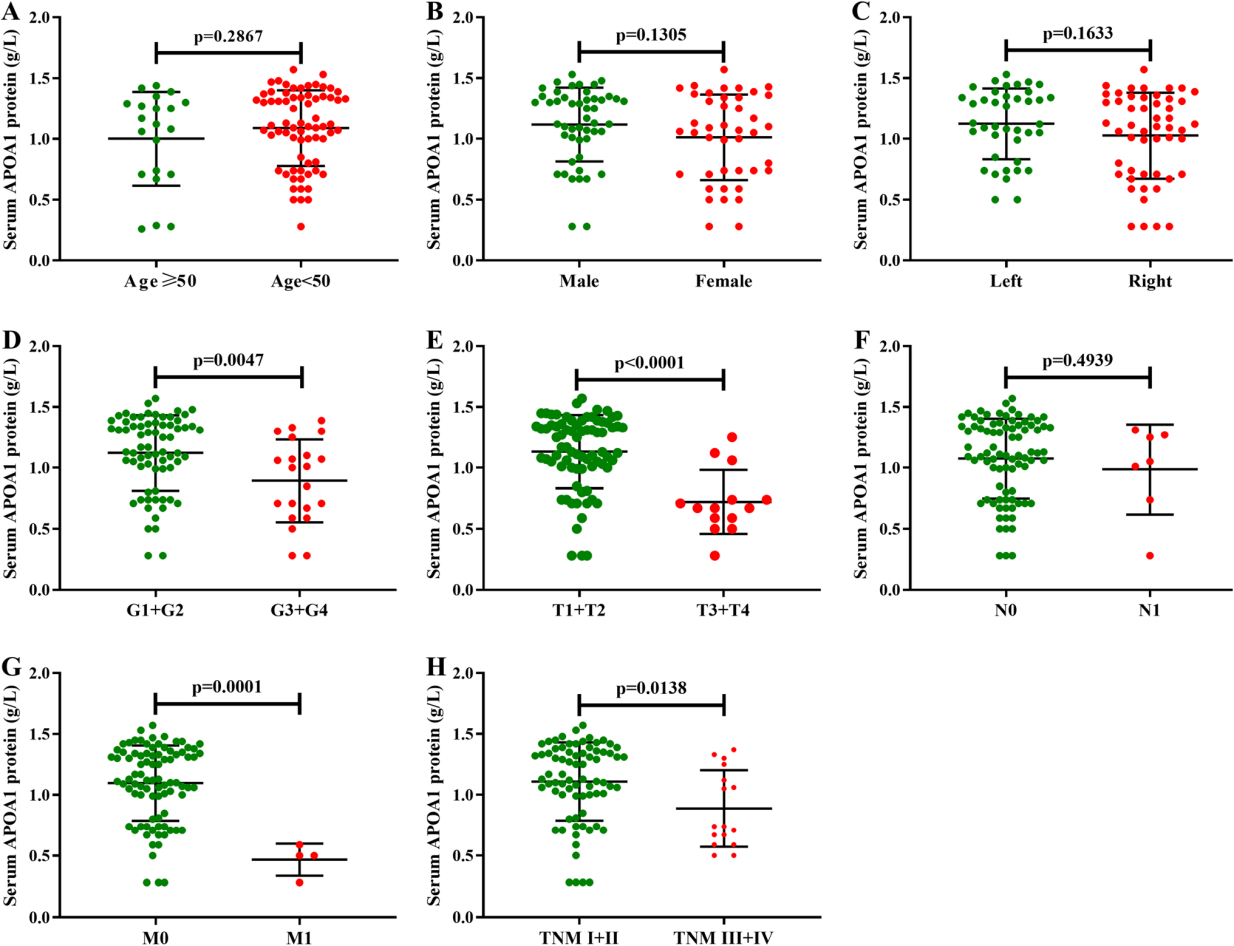
Relationships between levels of APOA1 protein and common clinical features in 91cases of KIRC patients by Grahpad Prism version 9.0 (https://www.graphpad.com/scientific-software/prism/). (**A**) age (*p* = 0.2867); (**B**) gender (*p* = 0.1305); (**C**) tumor site (*p* = 0.1633); (**D**) histological grade (*p* = 0.0047); (**E**) T stage (*p* < 0.0001); (**F**) N stage (*p* = 0.4939); (**G**) M stage (*p* = 0.0001); (**H**) TNM stage (*p* = 0.0138).

We collected clinical data from the RHWU cohort to explore the clinical value and prognostic significance of serum APOA1 protein levels in patients with KIRC. The findings suggested that concentrations of serum APOA1 protein levels were markedly decreased in patients with KIRC compared to healthy controls (*p* < 0.01) (Fig. 4A). ROC analysis showed that serum APOA1 protein levels exhibited a good diagnostic ability (AUC, 0.707; 95% CI 0.644–0.790) to differentiate patients with KIRC from healthy controls (Fig. 4B). Furthermore, serum APOA1 protein levels were significantly associated with a panel of clinical characteristics (Fig. 5), including histological grade (*p* = 0.0047), T stage (*p* < 0.0001), M stage (*p* = 0.0001), and TNM stage (*p* = 0.0138). The Kaplan–Meier plot revealed a close correlation between low levels of serum APOA1 protein and unfavorable OS (HR 2.84, *p* = 0.0407) in 91 patients with KIRC (Fig. 4C). Multivariate Cox analysis indicated that serum APOA1 protein level was a reliable index to predict OS (HR 2.91; *p* = 0.023) in patients with KIRC (Fig. 4D).

### Enrichment analysis

**Figure 6:**
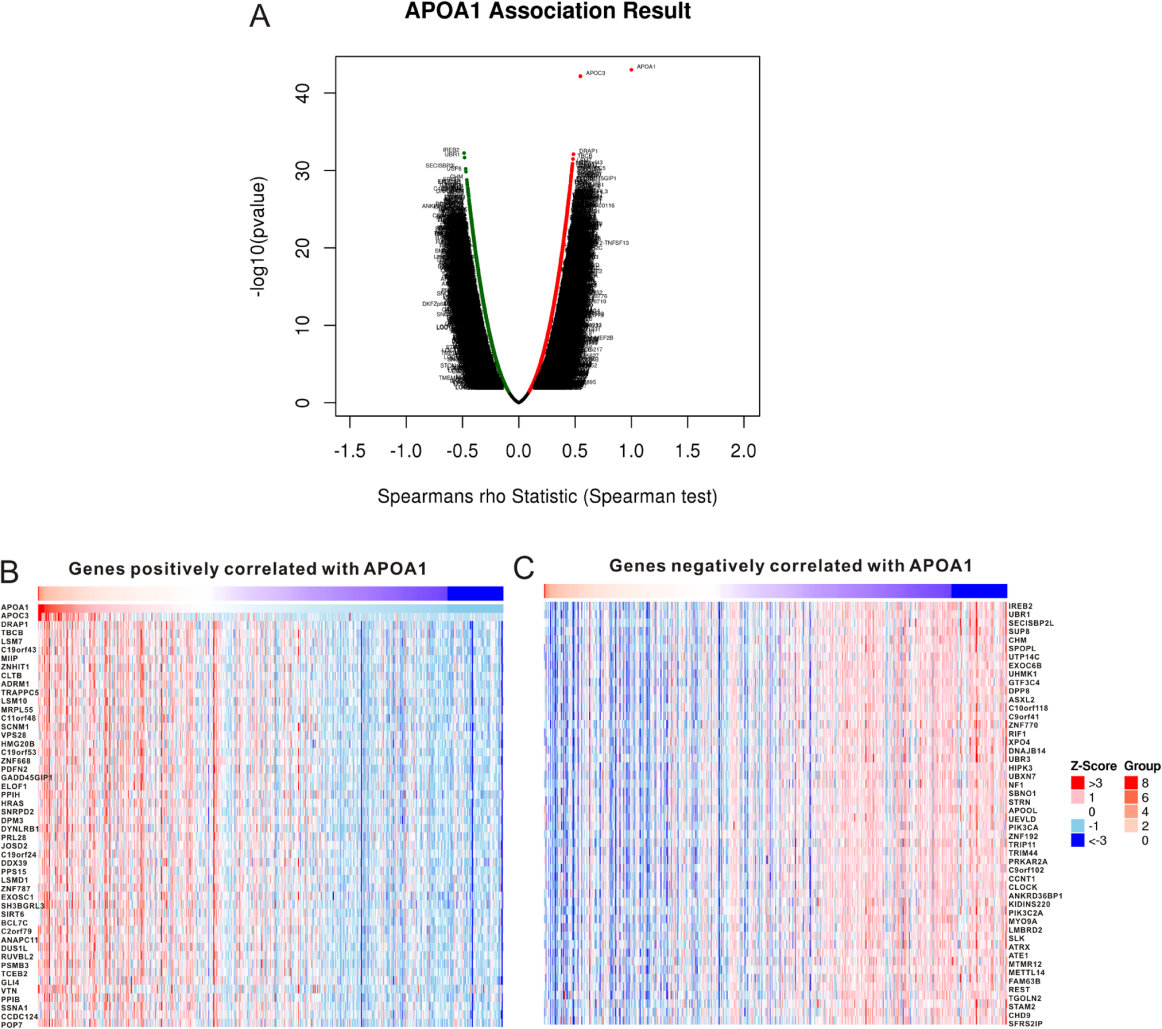
Co-expressed genes with APOA1 and Go analysis of these genes in KIRC based on the LinkedOmics database (http://www.linkedomics.org/). (**A**) Volcano plot of the genes relevant to APOA1 expression in KIRC tissues. (**B**) Significant genes positively correlated with APOA1 expression. (**C**) Significant genes negatively correlated with APOA1 expression.

The LinkedOmics database was used to identify the genes most significantly correlated with APOA1 expression in KIRC. The volcano map showed that all genes were correlated with APOA1 expression (Fig. 6A). The positively correlated genes are displayed in Fig. 6B and the negatively associated genes are listed in Fig. 6C. Subsequently, we utilized the GSEA module from the LinkedOmics database for enrichment analysis. The GO analysis (Table S3) demonstrated that the genes co-expressed with APOA1 were mostly implicated in mitochondrial gene expression, humoral immune response, protein targeting, ncRNA processing, and the nucleoside monophosphate metabolic process, among others. A KEGG analysis was then performed, demonstrating that these genes were primarily involved in the phosphatidylinositol signaling system, thyroid hormone signaling pathway, FoxO signaling pathway, purine metabolism, spliceosome, drug metabolism, and systemic lupus erythematosus. A detailed description of the KEGG analysis is shown in Table S4.

### Correlation between APOA1 and immune cells

After APOA1’s involvement in the human immune response was revealed via enrichment analysis, we searched the TIMER database to reveal any correlation between APOA1 expression and infiltrating immune cells and tumor purity. APOA1 expression was negatively correlated with tumor purity (r = − 0.152, *p* = 1.04e^−3^), CD8+ T cells (r = − 0.126, *p* = 8.51e^−3^), macrophages (r = − 0.144, *p* = 2.19e^−3^), neutrophils (r = − 0.164, *p* = 4.31e^−3^), and dendritic cells (r = − 0.101, *p* = 3.17e^−3^) (Fig. S3). No associations were observed between APOA1 expression and B cells or CD4+ T cells. We then used the correlation module to evaluate the correlation between APOA1 expression and gene markers of immune cell subtypes. We observed that APOA1 expression was correlated with B cells (CD19, CD79A) and Th2 cells (GATA3, STATA5A, and STATA6) (Table S5). We also determined the relationship between the expression of APOA1 mRNA in KIRC with PD-1 and PD-L1 due to the increasingly important role of immunotherapy in the treatment of renal cell carcinoma^24,25^. We observed a negative correlation between APOA1 expression and PD-L1 expression (r = − 0.283, *p* = 2.74e^−11^), while no association was observed between APOA1 and PD-1 (r = 0.085, *p* = 5.1e^−2^) (Fig. S4).

## Discussion

Renal cancer is a common malignancy in both men and women, with a high incidence and mortality rate^26^. However, the clinical use of molecular biomarkers associated with the prognosis in KIRC is limited. Therefore, our study was designed to explore the clinical applicability of APOA1, which is easily measurable in clinical practice, to predict survival in patients with KIRC. This study revealed that the expression of APOA1 mRNA is closely related to a series of clinical characteristics. Moreover, survival analyses showed that low expression of APOA1 mRNA was an independent indicator of favorable OS and DFS in patients with KIRC. The expression of the APOA1 protein was lower in KIRC tissue than in normal renal tissue. We found a similar trend in clinical data, with low levels of serum APOA1 protein associated with longer OS in patients with KIRC. These results indicate that APOA1 may be a promising biomarker to predict survival in patients with KIRC.

APOA1 is a major protein component of high-density lipoprotein^27^ and has been reported to be involved in angiogenesis, tumor growth, and metastasis^28,29^. There are several types of single-nucleotide polymorphisms in the *APOA1* gene, and an increased risk of renal cancer has been associated with the APOA1-75 A allele and the APOA1-75 AA genotype^30^. Furthermore, APOA1 has been correlated with survival in cancer patients. Luo et al.^31^ reported APOA1 as a novel prognostic index for risk definition in non-metastatic nasopharyngeal carcinoma. A recent clinical study, which included 144 patients with colorectal cancer, revealed that low levels of serum APOA1 protein were associated with systemic inflammation, advanced stage, and unfavorable survival^11^. Wang et al.^32^ suggested that APOA1 protein levels should be assessed pre-treatment as a predictor of OS in patients with esophageal squamous cell carcinoma. To our knowledge, this is the first study to uncover the association between low levels of APOA1 mRNA and hypomethylation of APOA1 with favorable survival in patients with KIRC. This study also analyzed the relationship between serum APOA1 levels and prognosis in patients with KIRC. Since APOA1 protein levels can be easily measured in the blood plasma, increased levels could be used as a biomarker of favorable survival in patients with KIRC.

We observed that APOA1 mRNA expression was statistically lower in the 535 KIRC tissue samples than in the 117 normal tissue samples. Regarding the distribution of APOA1 DNA methylation, we observed that the methylation levels of four important CpG sites (cg26734040, cg19324627, cg03010018, and cg24984312) were much higher in the KIRC tissue than in normal specimens. Therefore, we speculated that DNA methylation of APOA1 could partially explain the low expression of APOA1 mRNA in KIRC tissues. However, we noticed the discrepancy between favorable overall survival for the APOA1gene expression and worse OS for hypermethylation. As listed in Fig. 2C, we did not observe the negative association between APOA1 mRNA and DNA methylation, and there might be no relationship between APOA1 mRNA and DNA methylation in KIRC tissues. We also noticed that methylation level of APOA1 is significantly lower in KIRC tissues than that in normal tissues (Figure S5). Zheng et al^33^ reported that hypermethylation in the region of CpG islands promotes metastasis and is correlated with poorer survival outcomes in individuals with renal cancer. Hence, it’s no surprise that hypermethylation of APOA1 is correlated with poorer survival outcome in KIRC patients. Subsequently, we searched the HPA database to determine whether the expression of the APOA1 protein between KIRC and normal renal tissues differed. When immunohistochemical staining was performed, we found that the APOA1 protein was strongly stained in normal renal tissues, while weakly stained in KIRC tissues. Finally, we collected clinical data from our hospital to determine the differential expression of serum APOA1 protein. We observed that the levels of serum APOA1 protein were lower in patients with KIRC than in healthy controls. Therefore, we conservatively concluded that APOA1 might act as a potential tumor suppressor gene in KIRC.

Renal cancer is an immunogenic carcinoma associated with immune cell dysfunction and subsequent inability to control tumor growth^34^. Furthermore, one study found that APOA1 could inhibit monocyte recruitment and macrophage chemotaxis and therefore be involved in the immune response^35^. Mao et al.^36^ discovered that APOA1 overexpression could significantly reduce the COX-2-induced inflammatory response in hepatocytes. When we analyzed the genes co-expressed with APOA1 in the GSEA analysis, we found that APOA1 also participates in the human immune response in KIRC tissues. Therefore, we searched the TIMER database to analyze the association between APOA1 and immune infiltrates in KIRC. APOA1 expression was negatively correlated with an abundance of macrophages, CD8+ T cells, neutrophils, and dendritic cells. Due to the close association between APOA1 and immune cells in KIRC, we further studied the association between APOA1 and PD1/PD-L1 to determine whether APOA1 could be a target of immunotherapy for KIRC. We observed a negative correlation between APOA1 expression and PD-L1 expression, while no association was observed between APOA1 and PD-1. Previously, PD-L1 has been reported to act as an accurate biomarker for KIRC^37^, and the relationship between PD-L1 and survival was more robust than that of PD-1^38^. Furthermore, agents targeting PD-L1 have had promising effects in renal cell carcinoma^34^. The inverse association between APOA1 expression and PD-L1 implies that APOA1 plays a tumor suppressor role in KIRC and may be a novel therapeutic target for KIRC.

APOA1 belongs to the family of apolipoproteins A1/A4/E and plays an essential role in lipid metabolism^39^. Cristina et al.^11^ found that the overexpression of APOA1 can reduce the malignant features of colorectal cancer by regulating cholesterol export and downregulating COX-2 expression. Therefore, apabetalone, a synthetic stimulator of APOA1, is recognized as an inhibitor against colorectal cancer. The authors concluded that APOA1 could be a new therapeutic target for the treatment of colorectal cancer by modulating lipid metabolism. APOA1 has also been implicated in the progression of renal cell cancer through the regulation of lipid metabolism^40^, and APOA1 inhibition may be effective for treating renal cell cancer. More studies of APOA1 stimulators, such as apabetalone, to treat renal cell cancer are warranted.

There are some limitations to this study. First, we were unable to validate the clinical and prognostic significance of APOA1 mRNA in another public dataset for technical reasons. Second, although we further verified the prognostic value of preoperative serum APOA1 protein levels using data from our hospital, we did not investigate the prognostic role of dynamic changes in serum APOA1 in patients with KIRC. Third, we focused on bioinformatic analysis and clinical data, and no biomedical experiments were implemented to understand the in-depth mechanism of APOA1 in the progression of KIRC and the development of metastases. Therefore, a series of functional experiments to elucidate the role of APOA1 in KIRC would be valuable in the future.

## Conclusions

We comprehensively investigated the expression and prognostic profiles of APOA1 mRNA and protein levels in KIRC. APOA1 protein levels showed excellent diagnostic capability. Our study provides novel insights to identify a prognostic index with great clinical utility and therapeutic targets for KIRC treatment.

## Supplementary Information

Supplementary Information.

## Data Availability

The original contributions presented in the study are included in the article/Supplementary Material.

## Abbreviations

KIRC: Kidney renal clear cell carcinoma
APOA1: Apolipoprotein A1
RHWU: Renmin Hospital of Wuhan University
TCGA: The Cancer Genomic Atlas
GSEA: Gene Set Enrichment Analysis
HPA: The Human Protein Atlas
OS: Overall survival
DFS: Disease-free survival
HR: Hazard ratio
95% CI: 95% confidence index
AUC: Area under the curve
