# Supplementary material for: APOA1 mRNA and protein in kidney renal clear cell carcinoma correlate with the disease outcome
Source: Sci Rep. 2022 Jul 20;12:12406. doi: 10.1038/s41598-022-16434-6 (PMC9300670; doi:10.1038/s41598-022-16434-6)
Supplement: Supplementary file 1 — Supplementary Information. [file 41598_2022_16434_MOESM1_ESM.doc]

**Table S1 Correlation analysis of the relationship between APOA1 mRNA expression and methylation of APOA1 DNA CpG sites**

| CpG site | APOA1 mRNA expression | |
| --- | --- | --- |
|  | R correlation | P value |
| cg14795231 | 0.069 | 0.200 |
| cg25987102 | -0.139 | 0.048 |
| cg03856801 | -0.026 | 0.645 |
| cg26734040 | 0.012 | 0.826 |
| cg19324627 | 0.104 | 0.064 |
| cg03010018 | 0.055 | 0.333 |
| cg24984312 | -0.008 | 0.891 |
| cg20200605 | 0.065 | 0.253 |
| cg19360562 | -0.016 | 0.781 |
| cg00142925 | 0.121 | 0.031 |
| cg10753889 | 0.059 | 0.295 |
| cg13090478 | -0.013 | 0.820 |
| cg03044513 | -0.077 | 0.171 |
| cg23193059 | -0.071 | 0.205 |
| cg19299755 | -0.141 | 0.012 |

**TABLE S2 Correlation between APOA1 mRNA /methylation and clinical features of 533 KIRC patients**

| Clinical features | | APOA1 expression | | P value | APOA1 methylation | | P value |
| --- | --- | --- | --- | --- | --- | --- | --- |
|  |  | Low | High |  | Low | High |  |
| Age | ≥50 | 129 | 142 | 0.244 | 87 | 83 | 0.611 |
|  | <50 | 140 | 126 |  | 72 | 77 |  |
| Gender | Male | 165 | 181 | 0.134 | 104 | 99 | 0.512 |
|  | Female | 104 | 87 |  | 55 | 61 |  |
| Laterality | Left | 129 | 124 | 0.695 | 67 | 81 | 0.129 |
|  | Right | 140 | 144 |  | 92 | 79 |  |
| Longest dimension | ≥1.5 | 92 | 96 | 0.198 | 54 | 65 | 0.199 |
|  | <1.5 | 133 | 108 |  | 67 | 58 |  |
| Histologic grade | G1+G2 | 138 | 114 | 0.042 | 82 | 66 | 0.065 |
|  | G3+G4 | 131 | 154 |  | 77 | 94 |  |
| T stage | T1+T2 | 178 | 166 | 0.307 | 107 | 97 | 0.215 |
|  | T3+T4 | 91 | 102 |  | 52 | 63 |  |
| N stage | N0+NX | 260 | 260 | 0.811 | 152 | 159 | 0.031 |
|  | N1 | 9 | 8 |  | 7 | 1 |  |
| M stage | M0+MX | 233 | 225 | 0.384 | 145 | 124 | 0.001 |
|  | M1 | 36 | 43 |  | 14 | 36 |  |
| TNM stage | I+II | 172 | 154 | 0.124 | 103 | 86 | 0.045 |
|  | III+IV | 97 | 114 |  | 56 | 74 |  |
| Radiation therapy | Yes | 1 | 1 | 0.998 | 0 | 2 | 0.157 |
|  | No | 268 | 267 |  | 159 | 158 |  |
| Neoadjuvant treatment | Yes | 9 | 9 | 0.994 | 3 | 5 | 0.479 |
|  | No | 260 | 259 |  | 156 | 155 |  |
| White blood cell | Low | 4 | 5 | 0.047 | 2 | 3 | 0.492 |
|  | Normal | 139 | 129 |  | 84 | 88 |  |
|  | Elevated | 66 | 98 |  | 46 | 36 |  |
| Hemoglobin | Low | 111 | 152 | 0.075 | 75 | 79 | 0.355 |
|  | Normal | 99 | 87 |  | 58 | 53 |  |
|  | Elevated | 3 | 2 |  | 4 | 1 |  |
| Serum calcium | Low | 86 | 118 | 0.371 | 56 | 56 | 0.905 |
|  | Normal | 74 | 77 |  | 49 | 47 |  |
|  | Elevated | 4 | 6 |  | 3 | 2 |  |
| APOA1 expression | Low | - | - |  | 65 | 77 | 0.193 |
|  | High | - | - |  | 94 | 83 |  |
| APOA1 methylation | Low | 65 | 94 | 0.193 |  |  |  |
|  | High | 77 | 83 |  | - | - |  |

TABLE S3. Go analysis of APOA1 co-expressed genes in TCGA-KIRC dataset

| Gene set | Biological pathway | Representative genes |
| --- | --- | --- |
| GO_0140053 | mitochondrial gene expression | MRPL55 GADD45GIP1 MRPL23 HSD17B10 MRPS15 MRPS26 AURKAIP1 MRPL53 CHCHD1 MRPL52 |
| GO_0006959 | humoral immune response | VTN ROMO1 RPS19 FAM3A HPX HRG PSMB10 RPL30 C8B ZP3 |
| GO_0034470 | ncRNA processing | RPS15 EXOSC1 DUS1L POP7 RPP21 RPS19 LAGE3 DDX49 HSD17B10 EXOSC4 |
| GO_0006605 | protein targeting | HRAS RPL28 RPS15 PEX16 ROMO1 RPS19 ZDHHC24 TIMM17B PEX14 RPS20 |
| GO:0009123 | nucleoside monophosphate metabolic process | SIRT6 DGUOK EIF6 NDUFA3 BAD DTYMK NDUFB7 NDUFS5 GUK1 PARK7 |
| GO:0034504 | protein localization to nucleus | NF1 OSBPL8 NUP153 IPO8 CD2AP MED1 RANBP2 TOR1AIP2 NUP155 NUP133 |
| GO:0061919 | process utilizing autophagic mechanism | PIK3CA STAM2 MTM1 SMCR8 STX17 SPTLC2 PAFAH1B2 VPS13C RAB3GAP2 WDFY3 |
| GO:0018209 | peptidyl-serine modification | UHMK1 HIPK3 PIK3CA LATS1 FNIP2 TTBK2 LMTK2 BRAF PLCL2 MAPK8 |
| GO:0010256 | endomembrane system organization | USP8 TRIP11 STAM2 STX17 ARFGEF2 PAFAH1B1 CLCN3 RAB3GAP2 PIK3C3 ZW10 |
| GO:0000209 | protein polyubiquitination | TRIM44 UBE3A RC3H2 SHPRH ARIH1 ZFP91 UBR2 RNF111 RLIM FBXW11 |

**Table S4.** KEGG pathways of APOA1 co-expressed genes in KIRC

| Gene Set | Description | Size | Leading Edge Number | ES | NES | P Value | FDR |
| --- | --- | --- | --- | --- | --- | --- | --- |
| hsa04070 | Phosphatidylinositol signaling system | 97 | 38 | -0.4785 | -1.6302 | 0 | 0.17184 |
| hsa04919 | Thyroid hormone signaling pathway | 116 | 39 | -0.4681 | -1.615 | 0 | 0.16349 |
| hsa04068 | FoxO signaling pathway | 129 | 43 | -0.45779 | -1.5898 | 0 | 0.18244 |
| hsa00230 | Purine metabolism | 158 | 35 | 0.44598 | 1.526 | 0 | 0.057297 |
| hsa03040 | Spliceosome | 115 | 32 | 0.48796 | 1.6038 | 0 | 0.030406 |
| hsa00983 | Drug metabolism | 76 | 26 | 0.55257 | 1.6791 | 0 | 0.012543 |
| hsa05322 | Systemic lupus erythematosus | 122 | 52 | 0.51844 | 1.7172 | 0 | 0.0090231 |

**Table S5** Correlation analysis between APOA1 and relate genes and markers of immune cells in TIMER.

| Description | Gene markers |  |  |  |  |
| --- | --- | --- | --- | --- | --- |
|  |  | None | | Purity | |
|  |  | Correlation | P value | Correlation | P value |
| B cell | CD19 | 0.188 | 1.31e-05 | 0.152 | 1.08e-03 |
|  | CD79A | 0.185 | 1.68e-05 | 0.145 | 1.77e-03 |
| CD8+ T cell | CD8A | 0.029 | 5.07e-01 | -0.019 | 6.89e-01 |
|  | CD8B | 0.073 | 9.02e-02 | 0.032 | 4.99e-01 |
| Neutrophils | CD66b(CEACAM8) | -0.138 | 1.39e-03 | -0.129 | 5.68e-03 |
|  | CCR7 | 0.05 | 2.49e-01 | 0.006 | 8.99e-01 |
| Monocyte | CD86 | -0.079 | 7.01e-02 | -0.125 | 7.06e-03 |
| TAM | CCL2 | 0.003 | 9.41e-01 | -0.037 | 4.32e-01 |
|  | CD68 | -0.057 | 1.78e-01 | -0.074 | 1.11e-01 |
| Natural kill cell | KIR2DL1 | 0.042 | 3.29e-01 | 0.028 | 5.48e-01 |
|  | KIR2DL3 | 0.019 | 6.64e-01 | 0.003 | 9.49e-01 |
|  | KIR2DL4 | 0.084 | 5.34e-02 | 0.046 | 3.25e-01 |
|  | KIR3DL1 | 0.029 | 5.09e-01 | 0.015 | 7.42e-01 |
|  | KIR3DL2 | 0.08 | 6.6e-02 | 0.071 | 1.26e-01 |
|  | KIR3DL3 | 0.02 | 6.48e-01 | 0.014 | 7.72e-01 |
| Th1 | T-bet (TBX21) | 0.048 | 2.66e-01 | 0.021 | 6.46e-01 |
|  | STAT4 | 0.019 | 6.6e-01 | -0.014 | 7.58e-01 |
|  | STAT1 | -0.182 | 2.38e-05 | -0.226 | 8.96e-07 |
| Th2 | GATA3 | 0.101 | 1.97e-02 | -0.244 | 1.09e-07 |
|  | STAT6 | -0.179 | 3.39e-05 | -0.152 | 1.06e-03 |
|  | STAT5A | -0.034 | 4.32e-01 | -0.083 | 7.43e-02 |
| Th17 | IL17A | 0.066 | 1.28e-01 | 0.038 | 4.10e-01 |
| Tfh | BCL6 | -0.075 | 8.45e-02 | -0.079 | 8.95e-02 |
|  | IL21 | 0.037 | 3.99e-01 | 0.015 | 7.55e-01 |

TAM, Tumor associated macrophage; Th, T helper cell; Tfh, Follicular helper.


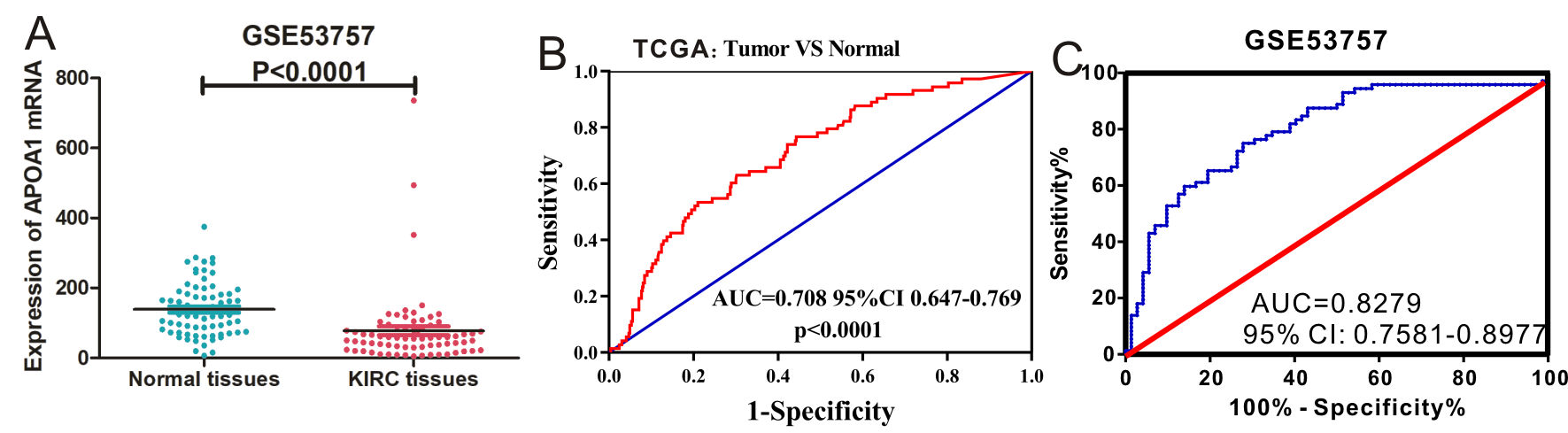


**Figure S1**. The expression of APOA1 mRNA in GSE53757 (**A**), and its diagnostic significance of APOA1 mRNA in KIRC based on TCGA-KIRC (**B**) and GSE53755 dataset (**C**) by Grahpad Prism version 9.0 (https://www.graphpad.com/scientific-software/prism/).


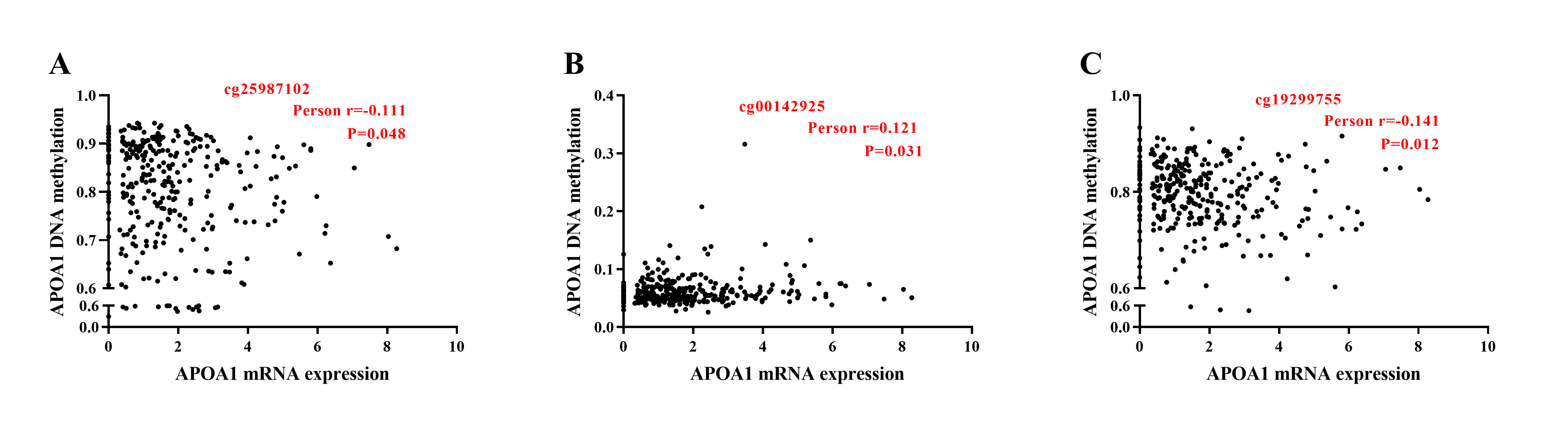


**Figure S2**. Correlation analysis between APOA1 expression and CpG sites of APOA1 DNA promoter by Grahpad Prism version 9.0 (https://www.graphpad.com/scientific-software/prism/). **A.** cg25987102. **B.** cg00142925. **C.** cg 19299755.


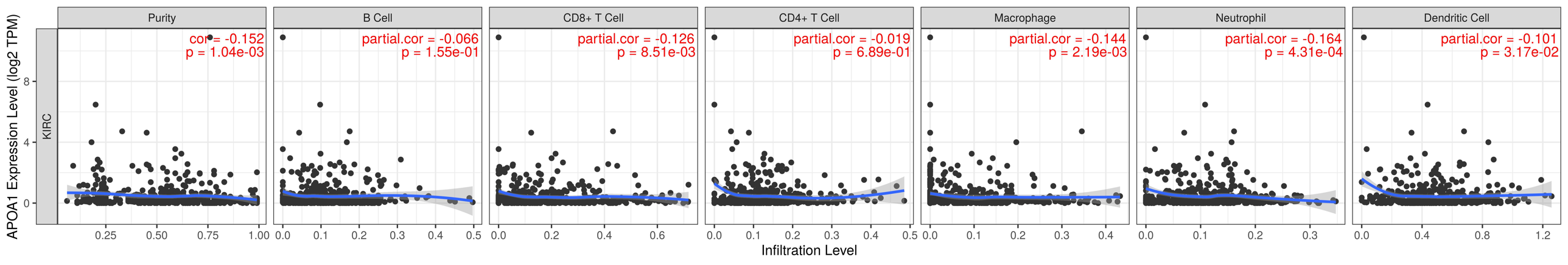


**Figure S3**. Association between APOA1 expression and a panel of immune cells in KIRC tissues by TIMER database (https://cistrome.shinyapps.io/timer/).


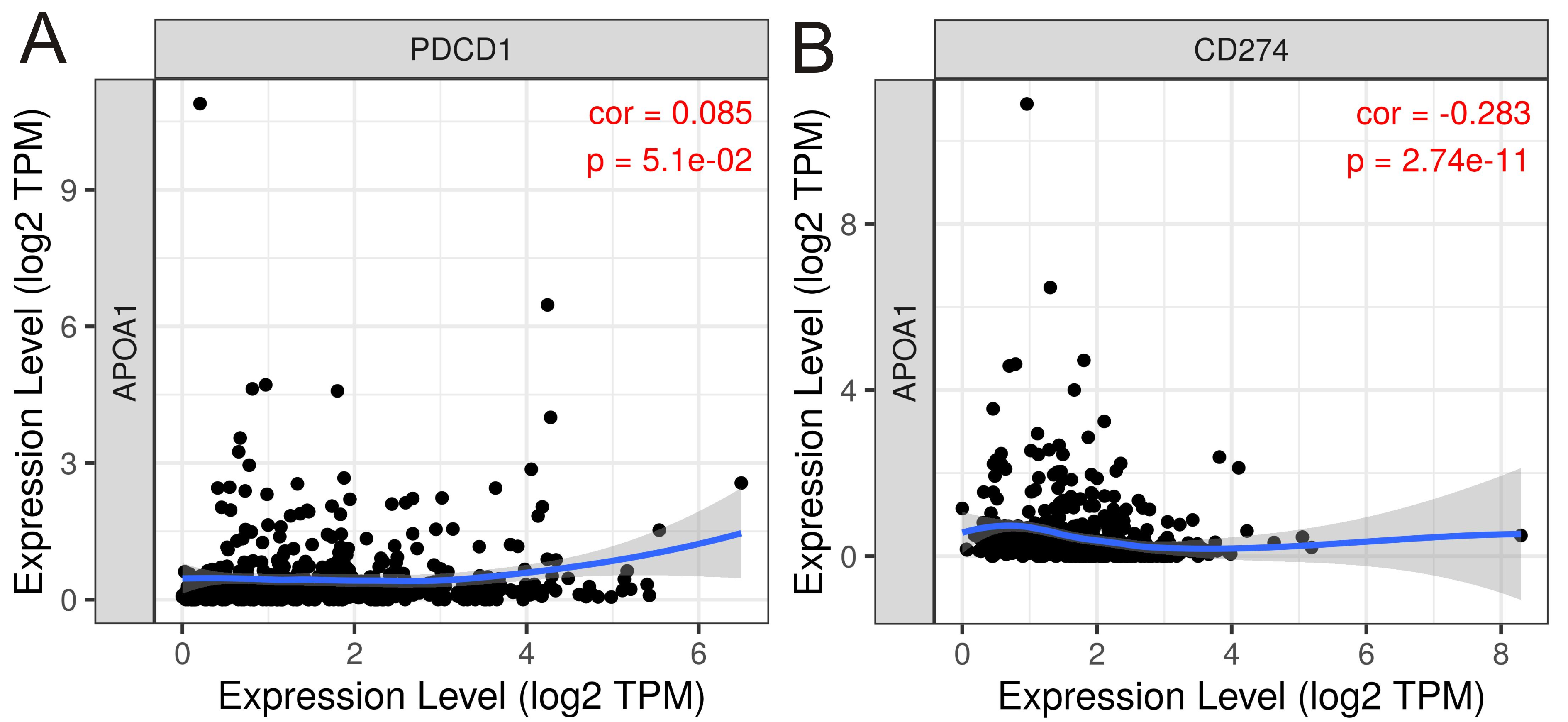


**Figure S4**. Correlation of APOA1 expression with levels of PD1 and PD-L1 in KIRC by TIMER database (https://cistrome.shinyapps.io/timer/). **A**. PD1, **B**. PD-L1.


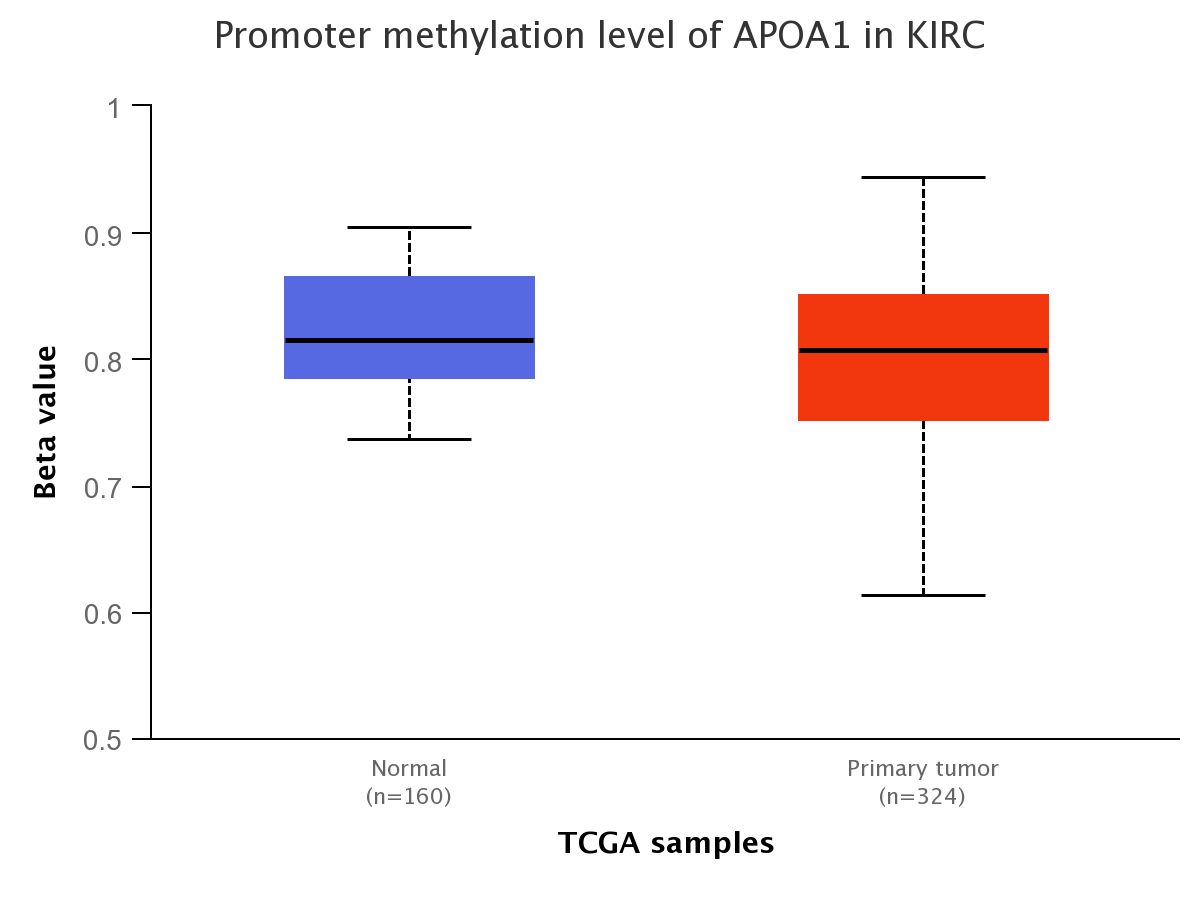


**Figure S5.** Promoter methylation level of APOA1 in KIRC tissues and the normal renal tissues from UALCAN (http://ualcan.path.uab.edu/index.html).
